# Time trends in body height according to educational level. A descriptive study from the Tromsø Study 1979–2016

**DOI:** 10.1371/journal.pone.0279965

**Published:** 2023-01-25

**Authors:** Sondre Haakonson Arntsen, Kristin Benjaminsen Borch, Tom Wilsgaard, Inger Njølstad, Anne Helen Hansen

**Affiliations:** 1 Department of Community Medicine, Faculty of Health Sciences, UiT The Arctic University of Norway, Tromsø, Norway; 2 University Hospital of North Norway, Tromsø, Norway; University of Jyvaskyla, FINLAND

## Abstract

**Objectives:**

The objective of our study was to describe time trends in body height according to attained educational level in women and men in Norway.

**Methods:**

We used previously collected data from six repeated cross-sectional studies in the population based Tromsø Study 1979–2016. Measured body height in cm and self-reported educational level were the primary outcome measures. We included 31 466 women and men aged 30–49 years, born between 1930 and 1977. Participants were stratified by 10-year birth cohorts and allocated into four groups based on attained levels of education. Descriptive statistics was used to estimate mean body height and calculate height differences between groups with different educational levels.

**Results:**

Mean body height increased by 3.4 cm (95% confidence interval (CI) 3.0, 3.8) in women (162.5–165.9 cm) and men (175.9–179.3 cm) between 1930 and 1977. The height difference between groups with primary education compared to long tertiary education was 5.1 cm (95% CI 3.7, 6.5) in women (161.6–166.7 cm) and 4.3 cm (95% CI 3.3, 5.3) in men (175.0–179.3 cm) born in 1930–39. The height differences between these educational groups were reduced to 3.0 cm (95% CI 1.9, 4.1) in women (163.6–166.6 cm) and 2.0 cm (95% CI 0.9, 3.1) in men (178.3–180.3 cm) born in 1970–77.

**Conclusions:**

Body height increased in women and men. Women and men with long tertiary education had the highest mean body height, which remained stable across all birth cohorts. Women and men in the three other groups had a gradual increase in height by birth cohort, reducing overall height differences between educational groups in our study population.

## Introduction

The maximal body height attained by an individual is a combined result of hereditary factors and social-economical-political-emotional (SEPE) conditions from conception to adulthood [1–6]. Several studies have shown an increase in height in European countries throughout the twentieth century, establishing Northern European populations as the tallest in the world [7–10]. This increase in height coincided with improvements in several areas influencing living conditions such as diet, educational level, income, working conditions, improved health services, and enhanced focus on public health [11, 12].

Throughout the twentieth century, body height increased in Norway, mirroring the secular trend in height found in other Nordic countries [9]. This trend has been described previously in young women and men in Norway, finding regional differences and a plateau in body height after 1960 [9, 13–15]. However, height trends in adult Norwegian women are not well described, since most studies on this subject in Norway have used data collected from male conscripts or children/adolescents [9, 13–15].

Concurrent with the secular trend in height, the proportion of Norwegians with tertiary education increased [16, 17]. Height differences between socioeconomic status (SES) groups based on educational level have been reported in several European countries, pointing towards a social inequality in body height [18–21]. A cross-sectional study of Norwegians born between 1926 and 1941, found that individuals with the longest education (17+ years) were 3.3 cm (men) and 3.2 cm (women) taller than individuals with the shortest education (7–9 years) [22].

However, there is a gap in the literature of recent and population based studies, describing time trends in measured body height across educational levels. Particularly, in adult women and other groups of men than conscripts/students. Data on body height according to educational level in Norwegian birth cohorts after 1941 is currently missing from the literature. The aim of the present study was to describe trends in body height in relation to attained educational level among women and men born between 1930 and 1977, using data from the population based Tromsø Study.

## Materials and methods

### Study design and setting

We used data from the population based Tromsø Study, conducted in the municipality of Tromsø, Northern Norway [23]. Tromsø is located around 400 km north of the Arctic Circle and is the largest municipality in Northern Norway with around 73 000 inhabitants in 2015. Inhabitants live in both rural and urban areas, with 80% of the population living in urban areas around the city of Tromsø [24]. This is comparable to the urban/rural distribution of the general Norwegian population, with 82.4% of Norwegians living in urban areas [25].

The Tromsø Study consists of seven surveys conducted between 1974 and 2016. The cohort profile and data collection have previously been described in detail [23, 24, 26, 27]. The first Tromsø Study (Tromsø1, 1974) comprised only of men, and was therefore not included in our study. In the consecutive studies (Tromsø2-7, 1979–2016), both women and men were invited [28]. Data were collected from clinical measurements and questionnaires.

### Study participants

From 1979 to 2016, a total of 59 722 residents were invited to participate, and 44 489 participated in one or more surveys of the Tromsø Study. In our study, participants were included after their first attendance, and we used their first measurements if they had attended more than one survey.

**Fig 1:**
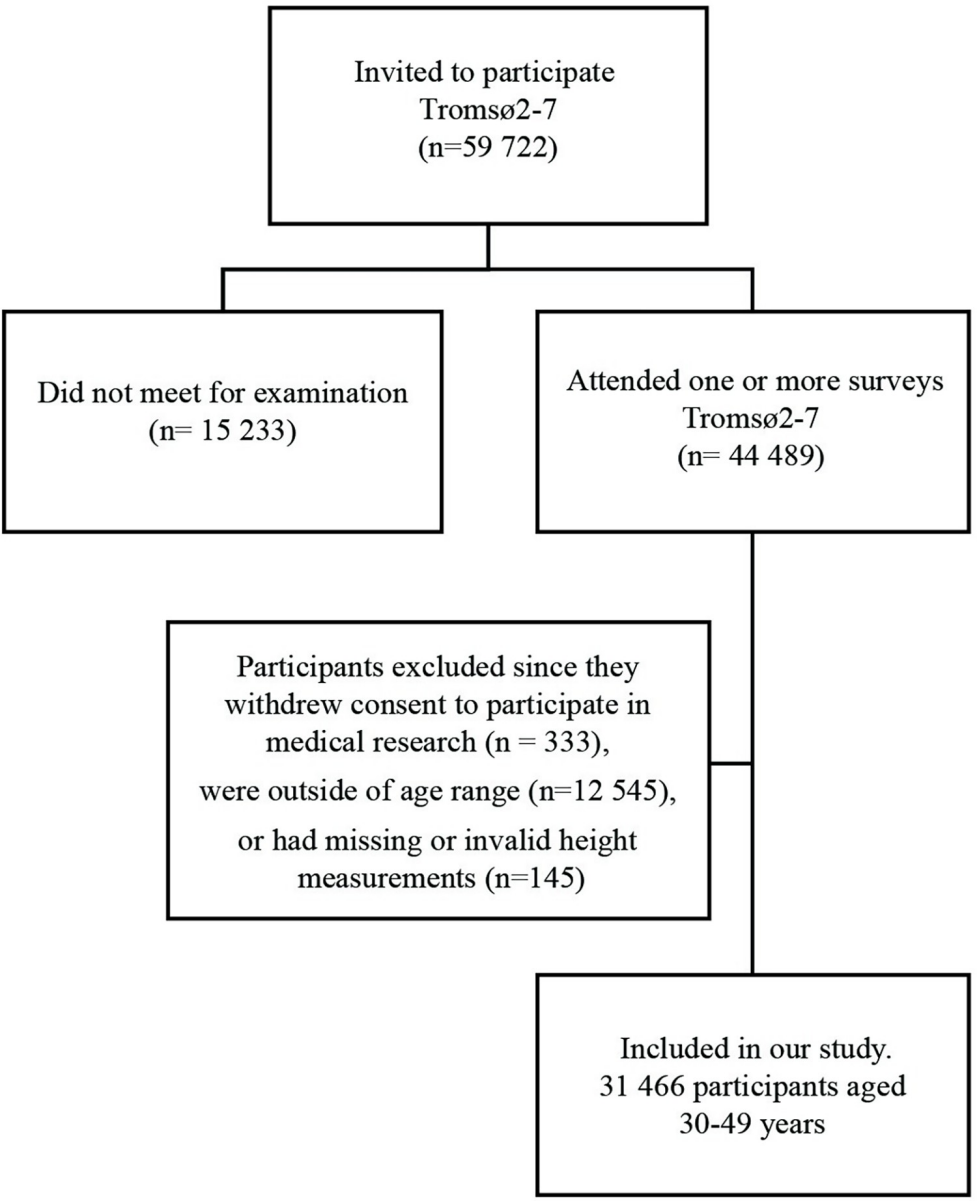
Flow chart of study population. The Tromsø Study 1979–2016. Tromsø2 (1979–1980), Tromsø3 (1986–1987), Tromsø4 (1994–1995), Tromsø5 (2001), Tromsø6 (2007–2008), Tromsø7 (2015–2016).

We excluded those who withdrew from Tromsø2-7 after data collection (n = 333), were outside of the age range (30–49 years) (n = 12 545) or had invalid height measurements (measured wearing shoes or headgear) (n = 145). The age range 30–49 years was chosen to ensure that participants had attained their highest level of education and to maximize the study sample size. Since the lower age limit for participation was 30 years in Tromsø5-6 and 40 years in Tromsø7. Participants aged 50+ years were excluded since height decline more rapidly past 50+ years [29]. The final study sample consisted of 31 466 participants (Fig 1).

### Variables

Data on age and sex were derived from the national 11-digit unique personal identification number provided to the Tromsø Study from the Norwegian National Population Registry. Body height and body weight were measured in a standing position without footwear. In Tromsø2-4, height was measured to the closest 1 cm with a wall-mounted ruler. Weight was measured to the closest 0.5 kg. In Tromsø5-7, height and weight were measured to the closest 0.1 cm and 0.1 kg, using an automatic electronic scale, Jenix DS 102 stadiometer (Dong Sahn Jenix, Seoul, Korea).

Data on educational level was collected from questionnaires. Tromsø2, Tromsø3 and Tromsø5 used open response categories. In Tromsø2 and Tromsø3, participants were asked, “How many years of education have you had”. In Tromsø4, participants were asked, “What is the highest educational level you have completed?” with five answer options (primary school and secondary school, vocational school, upper secondary school, high education less than 4 years, and high education 4 years or more). The question used in Tromsø5 was phrased “How many years of education have you completed”. Tromsø6 used the same question and response options as Tromsø4, while Tromsø7 used the same question as Tromsø4 together with four answering options (primary school and secondary school, upper secondary school, high education less than 4 years, and high education 4 years or more).

### Statistical analyses

Participants were grouped into 10-year birth cohorts. The educational categories were primary education (primary school and secondary school), secondary education (completed upper secondary school, ordinary or vocational subjects), short tertiary education (college/university < 4 years), and long tertiary education (college/university > 4 years).

We recoded years of education from Tromsø2,3 and 5 to categorical variables (up to 9 years of education = primary education, 10–12 years of education = secondary education, 13–15 years of education = short tertiary education, 16 + years of education = long tertiary education). Using descriptive statistics, we estimated mean body height and standard deviations (SD) in women and men. We calculated the difference (delta) in mean body height with 95% confidence interval (CI) between participants in different birth cohorts (born 1930–1939, 1940–1949, 1950–1959, 1960–1969, and 1970–1977). The same process was repeated according to sex and educational level. Statistical analyses were conducted using STATA version 17.0 (StataCorp., College Station, Texas, USA).

### Ethics

Participants in Tromsø4-7 gave written consent at the time of the data collection that the collected data could be used in future research. Participants from Tromsø2-3 provided verbal consent, which was common practice at the time of the data collection (Tromsø2 1979–80 and Tromsø3 1986–87). The use of data collected from participants who only provided verbal consent in Tromsø2-3 was approved by the Regional Committee for Medical and Health Research (REC North).

## Results

The anthropometric and educational characteristics in women and men according to birth cohort are presented in Table 1. Mean body height increased by 3.4 cm in women (95% CI 3.0, 3.8) and men (95% CI 3.0, 3.8) born in the 1970–77, compared to those born in the 1930–39 (Table 1). Among participants born in 1930–39, 4.9% of women and 9.8% of men had long tertiary education, while 67.8% of women and 55.8% of men had primary education.

In participants born in 1970–77, 46.3% of women and 34.0% of men had long tertiary education, 13.0% of women and 15.4% of men had primary education as their highest level of education (Table 1).

**Table 1:** Descriptive statistics according to sex and birth cohort*. The Tromsø Study2-7 (1979–2016).

	10-year birth cohort				
	1930–39	1940–49	1950–59	1960–69	1970–77
**Women**					
Number	1910	3800	4169	3887	2252
Age (years)	44.5 (3.0)	35.6 (4.3)	35.1 (3.7)	38.1 (6.25)	40.0 (5.0)
Body height (cm)	162.5 (6.0)	164.2 (6.2)	165.2 (6.2)	165.7 (6.24)	165.9 (6.5)
Body weight (kg)	63.3 (9.8)	61.7 (9.7)	63.1 (10.3)	68.8 (13.5)	72.5 (14.6)
BMI (kg/m^2^)	24.0 (3.6)	22.9 (3.4)	23.1 (3.5)	25.0 (4.70)	26.3 (5.1)
Primary education (%)	67.8	43.1	23.6	11.2	13.0
Secondary education (%)	19.8	28.4	35.6	40.8	20.2
Short tertiary education (%)	7.5	13.8	21.1	24.0	20.5
Long tertiary education (%)	4.9	14.8	19.7	24.0	46.3
**Men**					
Number	1964	4009	4014	3499	1966
Age (years)	44.5 (3.0)	36.0 (4.5)	35.4 (3.8)	38.5 (6.3)	40.4 (4.7)
Body height (cm)	175.9 (6.7)	177.4 (6.7)	178.4 (6.8)	179.1 (6.8)	179.3 (6.7)
Body weight (kg)	77.6 (10.5)	77.7 (10.6)	79.0 (11.3)	84.8 (13.9)	89.3 (14.7)
BMI (kg/m^2^)	25.1 (2.9)	24.7 (2.9)	24.8 (3.1)	26.4 (3.9)	27.8 (4.3)
Primary education (%)	55.8	32.7	22.5	15.9	15.4
Secondary education (%)	25.1	28.8	32.7	41.1	29.6
Short tertiary education (%)	9.4	16.6	22.3	21.2	21.1
Long tertiary education (%)	9.8	21.9	22.5	21.8	34.0

* Values are mean (standard deviation) or percent. Number = number of participants in each birth cohort. Primary education = primary school and secondary school/up to 9 years of education. Secondary education = upper secondary school/10-12 years of education. Short tertiary education = collage/university < 4 years. Long tertiary education = college/university > 4 years.

### Height and educational level in women

**Fig 2:**
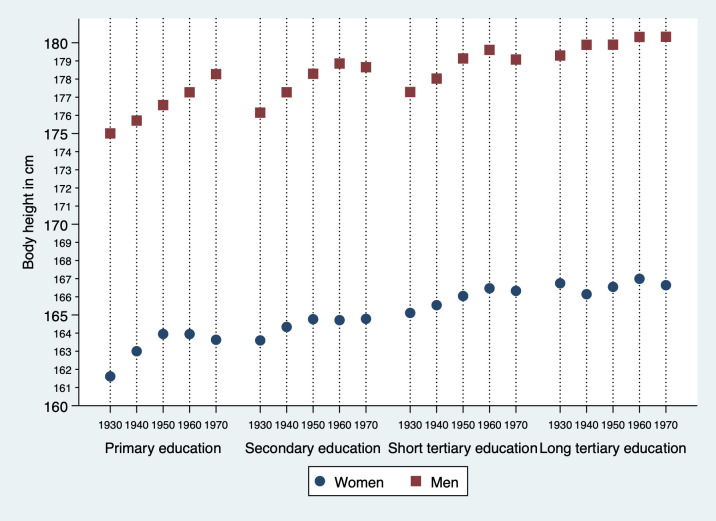
Trends in mean body height according to sex, educational level and 10-year birth cohort. The Tromsø Study2-7 (1979–2016). Primary education = primary school and secondary school, secondary education = upper secondary school, short tertiary education = college/university < 4 years, long tertiary education = college/university ≥ 4 years.

Body height in women according to birth cohort and attained educational level are presented in Table 2 and Fig 2. Body height remained stable (166.7–167.0 cm) in women with long tertiary education in all five birth cohorts, whereas in women with primary education, body height increased by 2.0 cm (95% CI 0.9, 3.1). An increased height by 1.2 cm was observed in women born in 1970–77 with secondary education or short tertiary education compared to women born in 1930–39 with secondary or short tertiary education, (95% CI 0.3, 3.3) (95% CI 0.0, 2.4), respectively. We observed a 5.1 cm (95% CI 3.7, 6.5) height difference between women with primary education compared to long tertiary education level in the 1930–39 birth cohort (Table 2). This difference between groups was reduced to 3.0 cm (95% CI 1.9, 4.1) in women born in 1970–77 (Table 2).

**Table 2:** Body height by birth cohort and educational level in women*. The Tromsø Study2-7 (1979–2016).

	10-year birth cohort					
	1930–39	1940–49	1950–59	1960–69	1970–77	Delta (95% CI)
Primary education						
Number	1124	1456	873	461	171	
Body height (cm)	161.6 (5.8)	163.0 (6.3)	164.0 (6.3)	164.0 (6.8)	163.6 (6.9)	2.0 (0.9, 3.1)
Secondary education						
Number	326	942	1404	1373	481	
Body height (cm)	163.6 (5.8)	164.3 (5.8)	164.5 (5.9)	164.7 (6.2)	164.8 (6.5)	1.2 (0.3, 3.3)
Short tertiary education						
Number	125	462	788	851	497	
Body height (cm)	165.1 (5.8)	165.5 (5.8)	166.0 (6.2)	166.2 (5.8)	166.3 (6.3)	1.2 (0.0, 2.4)
Long tertiary education						
Number	82	498	899	1184	1087	
Body height (cm)	166.7 (6.5)	166.1 (5.9)	166.3 (5.9)	167.0 (6.0)	166.6 (6.3)	-0.1 (-1.5, 1.4)

CI, confidence interval; Delta, mean difference between 1930–39 and 1970–77 birth cohort.

* Values are mean (standard deviation). Number = number of participants in each birth cohort. Primary education = primary school and secondary school, secondary education = upper secondary school, short tertiary education = college/university < 4 years, long tertiary education = college/university > 4 years.

### Body height and educational level in men

Body height in men according to birth cohort and education are presented in Table 3 and Fig 2. In men born 1970–77 with long tertiary education, body height was on average 1.0 cm (95% CI -0.1, 2.1) taller compared with men born 1930–39. We observed increased height in the three other educational groups as well, with the largest increase between cohorts (3.3 cm 95% CI 2.3, 4.4) among participants with primary education (Table 3).

**Table 3:** Mean body height in cm by birth cohort and educational level in men. The Tromsø Study2-7 (1979–2016).

	10-year birth cohort					
	1930–39	1940–49	1950–59	1960–69	1970–77	Delta (95% CI)
Primary education						
Number	957	1140	819	578	199	
Body height (cm)	175.0 (6.7)	175.7 (6.5)	176.6 (6.5)	177.3 (6.9)	178.3 (6.9)	3.3 (2.3, 4.4)
Secondary education						
Number	437	1013	1250	1309	607	
Body height (cm)	176.2 (6.6)	177.3 (6.8)	178.3 (6.8)	178.9 (6.7)	178.7 (6.4)	2.5 (1.7, 3.3)
Short tertiary education						
Number	162	579	826	697	445	
Body height (cm)	177.3 (6.4)	178.0 (6.3)	179.1 (6.7)	179.6 (6.5)	179.1 (6.6)	1.8 (0.6, 3.0)
Long tertiary education						
Number	168	792	923	898	706	
Body height (cm)	179.3 (6.3)	179.9 (6.5)	179.9 (6.6)	180.3 (7.0)	180.3 (6.7)	1.0 (-0.1, 2.1)

CI, confidence interval; Delta, mean difference between 1930–39 and 1970–77 birth cohort

* Values are mean (standard deviation). Number = number of participants in each birth cohort. Primary education = primary school and secondary school, secondary education = upper secondary school, short tertiary education = college/university < 4 years, long tertiary education = college/university > 4 years.

In men born 1930–39, we observed a 4.3 cm (95% CI 3.3, 5.3) height difference in men with long tertiary education compared to men with primary education (Table 3). The group difference between primary and long tertiary education was reduced to 2.0 cm (95% CI 0.9, 3.1) in men born 1970–77 (Table 3). In men with secondary education or short tertiary education, the highest mean height was observed in the 1960–69 cohort.

However, the difference between the 1960–69 and 1970–77 cohorts was small. Secondary education had a 0.2 cm difference (95% CI -0.8, 0.4), short tertiary education had a 0.5 cm difference (95% CI -1.3, 0.3) (Table 3).

## Discussion

### Principal findings

Body height increased in each successive birth cohort in women and men born between 1930 and 1977 who participated in the Tromsø Study. Women and men with long tertiary education had the highest mean body height, which remained stable across all birth cohorts. Women and men in the three other educational groups had a gradual increase in height by birth cohort, reducing overall height differences between groups. Similar trends were found in other studies from Europe including Norway [18, 21, 22, 30].

The reduced height differences between educational groups might be the result of unmeasured improvements in SEPE conditions, influencing attained body height in all educational groups except for the groups with long tertiary education. A recent review suggested that SEPE factors are influential in determining adult height in addition to hereditary factors [6]. The SEPE environment changed substantially in Norway during the growth period of our population [31]. The improved SEPE environment was characterised by reduced poverty, and improved access to nutrition, sanitation, and healthcare [31]. Improvements in these factors are beneficial for growth and increase adult height in the population over time. This epidemiological transition may explain the observed birth cohort effect on height across all educational groups, except for the long tertiary education groups in our study.

Several studies point to the influence of educational level or SES of parents on height, finding that individuals with high SES parents were taller than the general population [32–34]. These studies may be seen in conjunction with other studies from Spain and Japan, showing that university students were taller than the general population in the same age-group in first decades of the twentieth century [35, 36]. Most students in this period were from high SES families, which might also have been the case for our participants with tertiary education in the first two birth cohorts. However, this is uncertain since we were missing data on parental educational level in our study population. The height gap between students and the general population was reduced throughout the century, concurrent with the SEPE improvements in society [35, 36]. This is in line with our findings of reduced height differences between educational groups.

A large study of Swedish men, born 1950–75 found that taller individuals were more likely to have tertiary education. The authors also reported that the relationship between height and tertiary education diminished with increasing birth-years [19]. This is also in line with our findings of reduced height differences between groups based on educational attainment in the later birth cohorts. In contrast to our findings, a study from Finland and Sweden did not find reduced body height differences between educational levels among individuals born 1920–69 [37].

Our findings point towards a birth cohort effect with increased height by successive birth cohorts. This is in line with findings from other European studies over the past century [7–10]. In our study, we observed that height reached a plateau in women and men born in 1960 or later. The levelling off in women born after 1960 has also been observed in Finland, Sweden and Denmark [7]. Similarly, it has been reported that height in men plateaued in the Netherlands and the Scandinavian countries after 1960 [8].

For participants born in the first three birth cohorts, the period of growth coincided with the Second World War and its aftermath, which might have influenced attained adult height. A Norwegian study reported that mean height for school children dropped between 1 and 5 cm during the war years (1940–1945). However, this was followed by a period of catch-up growth in the following years suggesting that the impact of the war, on adult height was negligible in these birth cohorts [15].

In our study population, there is a shift in attained educational level with the high percentages of participants with tertiary educations, born after 1950. This is likely attributed to the establishment of the University of Tromsø in 1972, in combination with the establishment of the Norwegian State Educational Loan Fund in 1947. The public sector in Norway provides tuition free tertiary education through public universities, and student loans to citizens through the State Educational Loan Fund.

### Strengths and limitations

The main strength of our study is the large and population based study sample of the Tromsø Study with data from six cross-sectional studies collected over five decades, as well as the high number of participants with anthropometric measurements and information from questionnaires. Our study is strengthened by the longitudinal data on height and educational level in women, which has been underrepresented in the literature. In addition, we present data on trends in measured height in men older than conscripts/students.

Our study also has some limitations. Family financial situation during childhood and the educational level of parents are confounding factors on the association between height and educational level. These variables were lacking in Tromsø2-5, and thus not included in our study. Our findings are limited to the mainly urban Tromsø population, which equals the Norwegian population in most aspects. Nevertheless, generalisation of our findings to rural populations in Norway must be made with caution.

## Conclusions

Our study describe trends in body height across educational groups over time and builds upon a previous study with a rural population from Norway [22]. It also adds to the existing literature, linking differences in height to educational level across birth cohorts seen in other European countries. The reduced differences in height between educational levels may be seen as a social equalisation of body height in our study population. We find that body height at population level continues to be an indicator of SES in an urban Norwegian population. Clinicians and health policy makers should be aware of the relationship between height in the population and SES factors since both are known risk factor for several non-communicable diseases [38–40].

## References

[1] Little MA. Evolutionary Strategies for Body Size. Front Endocrinol 2020;11:107. doi: 10.3389/fendo.2020.00107 32210916 PMC7075806

[2] Cole TJ. The secular trend in human physical growth: a biological view. Econ Hum Biol. 2003;1(2):161–8. doi: 10.1016/S1570-677X(02)00033-3 15463971

[3] Silventoinen K. Determinants of variation in adult body height. J Biosoc Sci. 2003;35(2):263–85. doi: 10.1017/s0021932003002633 12664962

[4] Tanner JM. Growth as a mirror of the condition of society: secular trends and class distinctions. Acta Paediatr Jpn. 1987;29(1):96–103. doi: 10.1111/j.1442-200x.1987.tb00015.x 3144856

[5] Perkins JM, Subramanian SV, Davey Smith G, Özaltin E. Adult height, nutrition, and population health. Nutr Rev. 2016;74(3):149–65. doi: 10.1093/nutrit/nuv105 26928678 PMC4892290

[6] Bogin B. Social-Economic-Political-Emotional (SEPE) factors regulate human growth. HBPH. 2021;1.

[7] NCD Risk Factor Collaboration (NCD-RisC). A century of trends in adult human height. Elife. 2016;5:e13410. doi: 10.7554/eLife.13410 27458798 PMC4961475

[8] Hatton TJ, Bray BE. Long run trends in the heights of European men, 19th-20th centuries. Econ Hum Biol. 2010;8(3):405–13. doi: 10.1016/j.ehb.2010.03.001 20399715

[9] Holmgren A, Niklasson A, Aronson AS, Sjöberg A, Lissner L, Albertsson-Wikland K. Nordic populations are still getting taller—secular changes in height from the 20th to 21st century. Acta Paediatr. 2019;108(7):1311–20. doi: 10.1111/apa.14683 30838690

[10] Fudvoye J, Parent AS. Secular trends in growth. Ann Endocrinol 2017;78(2):88–91. doi: 10.1016/j.ando.2017.04.003 28483361

[11] Steckel RH. Stature and the Standard of Living. J Econ Lit. 1995;33(4):1903–40.

[12] German A, Mesch G, Hochberg Z. People Are Taller in Countries With Better Environmental Conditions. Front Endocrinol (Lausanne). 2020;11:106. doi: 10.3389/fendo.2020.00106 32218770 PMC7078159

[13] Rybak A, Bents D, Krüger J, Groth D. The end of the secular trend in Norway: spatial trends in body height of Norwegian conscripts in the 19(th), 20(th) and 21(st) century. Anthropol Anz. 2020. 10.1127/anthranz/2020/1254 32588018

[14] Sunder M. The making of giants in a welfare state: the Norwegian experience in the 20th century. Econ Hum Biol. 2003;1(2):267–76. doi: 10.1016/S1570-677X(03)00040-6 15463978

[15] Brundtland GH, Liestøl K, Walløe L. Height, weight and menarcheal age of Oslo schoolchildren during the last 60 years. Ann Hum Biol. 1980;7(4):307–22. doi: 10.1080/03014468000004381 7436344

[16] Steingrímsdóttir OA, Næss Ø, Moe JO, Grøholt EK, Thelle DS, Strand BH, et al. Trends in life expectancy by education in Norway 1961–2009. Eur J Epidemiol. 2012;27(3):163–71. doi: 10.1007/s10654-012-9663-0 22392586

[17] Enroth L, Jasilionis D, Németh L, Strand BH, Tanjung I, Sundberg L, et al. Changes in socioeconomic differentials in old age life expectancy in four Nordic countries: the impact of educational expansion and education-specific mortality. Eur J Ageing. 2022;19(2):161–73. doi: 10.1007/s10433-022-00698-y 35663915 PMC9156635

[18] Cavelaars AE, Kunst AE, Geurts JJ, Crialesi R, Grötvedt L, Helmert U, et al. Persistent variations in average height between countries and between socio-economic groups: an overview of 10 European countries. Ann Hum Biol. 2000;27(4):407–21. doi: 10.1080/03014460050044883 10942348

[19] Magnusson PK, Rasmussen F, Gyllensten UB. Height at age 18 years is a strong predictor of attained education later in life: cohort study of over 950,000 Swedish men. Int J Epidemiol. 2006;35(3):658–63. doi: 10.1093/ije/dyl011 16446353

[20] Huang Y, van Poppel F, Lumey LH. Differences in height by education among 371,105 Dutch military conscripts. Econ Hum Biol. 2015;17:202–7. doi: 10.1016/j.ehb.2014.11.002 25487837 PMC8066435

[21] Mackenbach JP. Socio-economic health differences in The Netherlands: a review of recent empirical findings. Soc Sci Med. 1992;34(3):213–26. doi: 10.1016/0277-9536(92)90264-q 1557663

[22] Meyer HE, Selmer R. Income, educational level and body height. Ann Hum Biol. 1999;26(3):219–27. doi: 10.1080/030144699282723 10355493

[23] Njølstad I, Mathiesen EB, Schirmer H, Thelle DS. The Tromsø study 1974–2016: 40 years of cardiovascular research. Scand Cardiovasc J. 2016;50(5–6):276–81. 27650831 10.1080/14017431.2016.1239837

[24] Hopstock LA, Grimsgaard S, Johansen H, Kanstad K, Wilsgaard T, Eggen AE. The seventh survey of the Tromsø Study (Tromsø7) 2015–2016: study design, data collection, attendance, and prevalence of risk factors and disease in a multipurpose population-based health survey. Scand J Public Health. 2022:14034948221092294. 10.1177/14034948221092294 PMC9578102 35509230

[25] Statistics Norway. Population and land area in urban settlements 2021 [cited 2022 14. June]. Available from: https://www.ssb.no/en/befolkning/folketall/statistikk/tettsteders-befolkning-og-areal.

[26] Jacobsen BK, Eggen AE, Mathiesen EB, Wilsgaard T, Njølstad I. Cohort profile: the Tromso Study. Int J Epidemiol. 2012;41(4):961–7. doi: 10.1093/ije/dyr049 21422063 PMC3429870

[27] Eggen AE, Mathiesen EB, Wilsgaard T, Jacobsen BK, Njølstad I. The sixth survey of the Tromso Study (Tromso 6) in 2007–08: Collaborative research in the interface between clinical medicine and epidemiology: Study objectives, design, data collection procedures, and attendance in a multipurpose population-based health survey. Scand J Public Health. 2013;41(1):65–80. doi: 10.1177/1403494812469851 23341355

[28] The Tromsø Study UiT The Arctic University of Norway; [cited 2022 01. April]. Available from: https://uit.no/research/tromsostudy.

[29] Cline MG, Meredith KE, Boyer JT, Burrows B. Decline of Height with Age in Adults in a General Population Sample: Estimating Maximum Height and Distinguishing Birth Cohort Effects from Actual Loss of Stature with Aging. Hum Biol. 1989;61(3):415–25. 2807265

[30] Singh-Manoux A, Gourmelen J, Ferrie J, Silventoinen K, Guéguen A, Stringhini S, et al. Trends in the association between height and socioeconomic indicators in France, 1970–2003. Econ Hum Biol. 2010;8(3):396–404. doi: 10.1016/j.ehb.2010.03.002 20400383 PMC2914812

[31] Norwegian Institute of Public Health. Folkehelse i Norge 1814–2014 2014 [updated 7. Aug 2018; cited 2022 1. June]. Available from: https://www.fhi.no/nettpub/hin/folkehelse-i-historien/folkehelse-i-norge-1814—2014/.

[32] Galobardes B, McCormack VA, McCarron P, Howe LD, Lynch JW, Lawlor DA, et al. Social Inequalities in Height: Persisting Differences Today Depend upon Height of the Parents. PLoS ONE. 2012;7. doi: 10.1371/journal.pone.0029118 22238588 PMC3253075

[33] Webb E, Kuh D, Peasey A, Pajak A, Malyutina S, Kubinova R, et al. Childhood socioeconomic circumstances and adult height and leg length in central and eastern Europe. J Epidemiol Community Health. 2008;62(4):351–7. doi: 10.1136/jech.2006.056457 18339829

[34] Kuh D, Wadsworth M. Parental height: childhood environment and subsequent adult height in a national birth cohort. Int J Epidemiol. 1989;18(3):663–8. doi: 10.1093/ije/18.3.663 2807671

[35] Linares-Luján AM, Parejo-Moruno FM. Rural Height Penalty or Socioeconomic Penalization? The Nutritional Inequality in Backward Spain. Int J Environ Res Public Health. 2021;18(9). doi: 10.3390/ijerph18094483 33922549 PMC8122891

[36] Kouchi M. Secular change and socioeconomic difference in height in Japan. Anthropol Sci. 1996;104(4):325–40.

[37] Silventoinen K, Lahelma E, Lundberg O, Rahkonen O. Body height, birth cohort and social background in Finland and Sweden. Eur J Public Health. 2001;11(2):124–9. doi: 10.1093/eurpub/11.2.124 11420796

[38] Lai FY, Nath M, Hamby SE, Thompson JR, Nelson CP, Samani NJ. Adult height and risk of 50 diseases: a combined epidemiological and genetic analysis. BMC Med. 2018;16(1):187. doi: 10.1186/s12916-018-1175-7 30355295 PMC6201543

[39] Emerging Risk Factors Collaboration. Adult height and the risk of cause-specific death and vascular morbidity in 1 million people: individual participant meta-analysis. Int J Epidemiol. 2012;41(5):1419–33. doi: 10.1093/ije/dys086 22825588 PMC3465767

[40] Sommer I, Griebler U, Mahlknecht P, Thaler K, Bouskill K, Gartlehner G, et al. Socioeconomic inequalities in non-communicable diseases and their risk factors: an overview of systematic reviews. BMC Public Health. 2015;15(1):914. doi: 10.1186/s12889-015-2227-y 26385563 PMC4575459

